# Management of Audiological Disorders in Cochlear Implants: Outcomes in Demanding Listening Situations and Future Perspectives

**DOI:** 10.3390/jcm14062089

**Published:** 2025-03-19

**Authors:** Matthias Hey, Ulrich Hoppe

**Affiliations:** 1 Audiology, ENT Clinic, UKSH Kiel, 24105 Kiel, Germany; 2 Department of Audiology, ENT-Clinic, University of Erlangen-Nürnberg, 91054 Erlangen, Germany; ulrich.hoppe@uk-erlangen.de

## 1. Introduction

Severe to profound sensorineural hearing loss can nowadays successfully be treated by cochlear implantation. However, cochlear implant (CI) recipients still face communication challenges in several everyday communication situations.

In the early years of provision of deaf patients with a CI, the focus was initially on the perception of suprathreshold speech in quiet [1,2,3]. Success was defined as better speech understanding along with lip reading. There has been great progress over the last three decades concerning recognition of speech in quiet. Nowadays, the majority of cochlear implant recipients are scoring highly in open-set speech perception in quiet [4,5].

However, although CI recipients are able to perform well in quiet, they are still facing a significant disadvantage when noise is present. In noisy environments, speech understanding of CI recipients deteriorates rapidly as the level of background noise increases [5,6,7]. With further progress in signal processing in CI systems, suprathreshold speech intelligibility in noise was the next field of great research activities [5,6,7].

A common measure for the ability of a listener to understand speech in noise is the speech reception threshold (SRT), defined as the signal-to-noise ratio (SNR) where 50% of the speech items are correctly understood. Compared to normal-hearing listeners, the SRT achievable for CI recipients is typically much poorer [8,9,10].

Modern CI systems try to improve speech comprehension in a noisy situation by utilizing digital signal processing algorithms. A range of sophisticated signal processing algorithms for CI sound processors have been introduced such as conventional beamformer, dynamic range optimization, and spatial post-filter technologies. Each technical approach aimed to improve the speech perception in a specific listening situation like speech in quiet [11,12], speech in noise [13,14], and speech in spatially distributed noise [15,16,17]. Such noise suppression algorithms are especially successful in stationary noise. For these conditions, an additional significant improvement was found by bilateral/bimodal hearing systems [5,18,19]. The majority of the investigation was performed in stationary noise.

Today, CI systems are not only suitable for hearing and understanding in easy-to-understand dialog situations or in stationary noise. The latter is not representative of typical acoustic listening situations. Patients want to participate in everyday life where noises can be quite different. The ‘normal hearing tests’ in quiet or in stationary noise characterize everyday listening situations only to a certain extent. In order to describe these demands more precisely, the requirements for ecological validity are summarized as follows: “In hearing science, ecological validity refers to the degree to which research findings reflect real-life hearing-related function, activity, or participation” [20]. The idea of ecological validity is considered as a concept that investigates to what extent the result of a specific audiometric investigation is able to draw a relation to outcomes in hearing situations that can be found in everyday life. One aim of the methodological approach of ecological validity is to reduce the differences between the measurements in the laboratory and the everyday listening environment. This can be achieved by using more realistic acoustic test scenarios [20,21,22].

Nevertheless, speech comprehension of CI users in contrast to normal hearing listeners decreases when transferring stationary noisy situation to fluctuating noise types [9,10,23]. It must be considered that temporal fluctuations of the interfering noise diminish speech comprehension of the hearing impaired. In particular, patients struggle in the presence of fluctuating babble noise types, commonly found in environments such as schools, restaurants, parties, and shopping centers.

These situations are often associated with greater listening effort, which affects the concentration span for auditory perception and is not assessed by audiometric speech tests [24,25].

According to studies on datalogging in cochlear implant systems, the majority of speech components are in the range below 60 dB_SPL_ [17]. These levels relate to soft speech or speech from a distance. In most studies, the standard target value for understanding monosyllabic words is considered to be 65 or 70 dB_SPL_. The supplementary comprehension of softer speech below 65 dB has also been the subject of recent studies [26,27,28].

## 2. Conclusions

In summary, ongoing research aims to widen the number of CI users who have sufficient speech comprehension in quiet and in stationary noise in general [29]. Further development can be addressed especially in everyday listening situation as follows:

- Enhanced speech comprehension in temporal fluctuating noise, which may also incorporate spatially distributed noise sources;
- Reduced listening effort;
- Audiometric test procedures that better reflect everyday listening situations;
- Improved understanding of soft and distant speech.
